# PROM2 promotes gemcitabine chemoresistance via activating the Akt signaling pathway in pancreatic cancer

**DOI:** 10.1038/s12276-020-0390-4

**Published:** 2020-03-02

**Authors:** Wenbin Li, Yue Zhu, Kelin Zhang, Xianhuan Yu, Haoming Lin, Wenrui Wu, Yaorong Peng, Jian Sun

**Affiliations:** 1 Department of Hepatobiliary and pancreatic Surgery, Sun Yat-Sen Memorial Hospital, Sun Yat-Sen University, Guangzhou, 510120 China; 2 Guangdong Provincial Key Laboratory of Malignant Tumor Epigenetics and Gene Regulation, Medical Research Center, Sun Yat-Sen Memorial Hospital, Sun Yat-Sen University, Guangzhou, 510120 China; 3 Department of Vascular and Thyroid Surgery, Sun Yat-Sen Memorial Hospital, Sun Yat-Sen University, Guangzhou, 510120 China; 4 Department of Surgical Intensive Care Unit, Sun Yat‑sen Memorial Hospital, Sun Yat-sen University, Guangzhou, 510120 China

**Keywords:** Pancreatic cancer, Prognostic markers

## Abstract

In recent years, the deoxycytidine analogue gemcitabine (2′,2′,-difluorodeoxycytidine) has become the first-line chemotherapeutic agent for patients with pancreatic cancer. However, due to the intrinsic resistance of pancreatic cancer cells, gemcitabine-based chemotherapy yields limited disease control, with >85% disease progression at 6 months from diagnosis. Therefore, elucidating the mechanisms of chemoresistance is a critical step in improving cancer therapy, especially for the treatment of pancreatic cancer. We show PROM2, a transmembrane glycoprotein, is ubiquitously upregulated in pancreatic cancer cell. We also found higher PROM2 expression is associated with shortened overall and disease-free survival times in patients diagnosed with pancreatic cancer. We provide evidence that PROM2 promotes chemoresistance to gemcitabine both in vivo and in vitro. Mechanistically, we demonstrate that PROM2 could directly interacted with Akt and activates the Akt signaling pathway, which thus inhibiting gemcitabine-induced apoptosis. As further evidence, we show PROM2 expression and Akt phosphorylation both promote gemcitabine chemoresistance, and cause poorer survival in clinical samples with pancreatic cancer. Combining gemcitabine with the Akt inhibitor MK-2206 facilitated significant tumor shrinkage and dramatically elevated the survival status in mice xenografted with pancreatic cancer cells. Our findings not only establish PROM2 as a novel positive regulator of the Akt signaling pathway and a candidate prognostic indicator of gemcitabine response, but also provide a neo-therapeutic approach for patients resistant to gemcitabine treatment.

## Pancreatic cancer: Exploring a pathway to drug resistance

A cell membrane protein called PROM2 promotes the resistance of pancreatic cancer to the anti-cancer drug gemcitabine, suggesting PROM2 and the molecular signaling pathway it stimulates could be targeted by new treatments. Researchers in China led by Jian Sun at Sun Yat-Sen University, Guangzhou, investigated the role of PROM2 in cultured human pancreatic cancer cells and in a mouse model of pancreatic cancer. Production and activity of PROM2 were increased in cancer cells, leading to increased resistance to gemcitabine. The researchers found that PROM2’s promotion of gemcitabine resistance was linked to its ability to bind to another protein called Akt. This interaction stimulates the Akt signaling pathway, sustaining cancer cells. Combining gemcitabine therapy with an Akt pathway inhibitor restored cancer cell sensitivity to gemcitabine, revealing a potential approach to developing drugs to overcome gemcitabine resistance.

## Introduction

Pancreatic cancer ranks as the fourth leading cause of cancer-related death worldwide^1^ with a dismal 5-year survival rate of <7%^2–4^. In 2018, 458,918 new cases of pancreatic cancer were diagnosed, and there were 458,918 deaths globally^5^. Gemcitabine (2′,2′,-difluorodeoxycytidine) was approved in 1996 by FDA for pancreatic cancer, and has become the first-line chemotherapeutic agent for patients with advanced pancreatic cancer for more than two decades^6–8^. However, due to the intrinsic resistance of pancreatic cancer cells, gemcitabine-based chemotherapy yields limited disease control, with >85% disease progression at 6 months from diagnosis^9,10^. Pancreatic cancer is likely to become the second leading cause of cancer mortality in 2020, owing to the more advanced therapies for other cancers and increasing prevalence of pancreatic cancer worldwide^11,12^. Therefore, unveiling the regulatory mechanism underlying chemoresistance of gemcitabine in pancreatic cancer is of urgent need.

Deregulation of the Akt signaling pathway is a frequent occurrence in pancreatic cancer and is significantly correlated with gemcitabine chemoresistance^13,14^. Akt activity is tightly controlled: it is activated by growth factors or cellular stress in the cytoplasm, and then recruited to the plasma membrane where it is phosphorylated (at Thr308 and Ser473)^15–17^. Phosphorylated Akt then phosphorylates and inactivate BAD, a pro-apoptotic member of Bcl-2 family that initiates the late stages of apoptosis^18,19^. Activated Akt can also phosphorylate Caspase-9 and impair its function in the apoptotic cascade^20,21^. Above all, constructive activation of the Akt signaling pathway can protect cells from drug-induced apoptosis and contribute to chemoresistance^13,22,23^. Thus, identifying potential modulators of the Akt signaling pathway would be crucial in restraining gemcitabine chemoresistance.

The prominin proteins (PROM1 and PROM2) are vital members of the pentaspan transmembrane family that are enriched at plasma membrane protrusions. Indeed, both PROM1 and PROM2 have been reported to bind cholesterol directly and to associate with membrane microdomains in some cell types^24–26^. PROM1 (CD133) is widely known as a biological marker for cancer stem cells of certain cell types, and is present in epithelial and non-epithelial cells^27–29^. Meanwhile, PROM2 has been rarely studied, and its expression is restricted to epithelial cells^30,31^. It is proposed that PROM2 inhibits Cdc42 dependent fluid phase endocytosis in human skin fibroblasts and Chinese hamster ovary cells^32^. In addition, other studies have shown PROM2 is upregulated in lung cancer and chromophobe renal cell carcinoma^33,34^. However, the biological function of PROM2 has not yet been verified and its role in pancreatic cancer is unclear.

In this study, we aimed to unravel the potential role(s) of PROM2 in pancreatic cancer progression and development of chemoresistance to gemcitabine.

## Materials and methods

### Tissue specimens and patient information

**Fig. 1:**
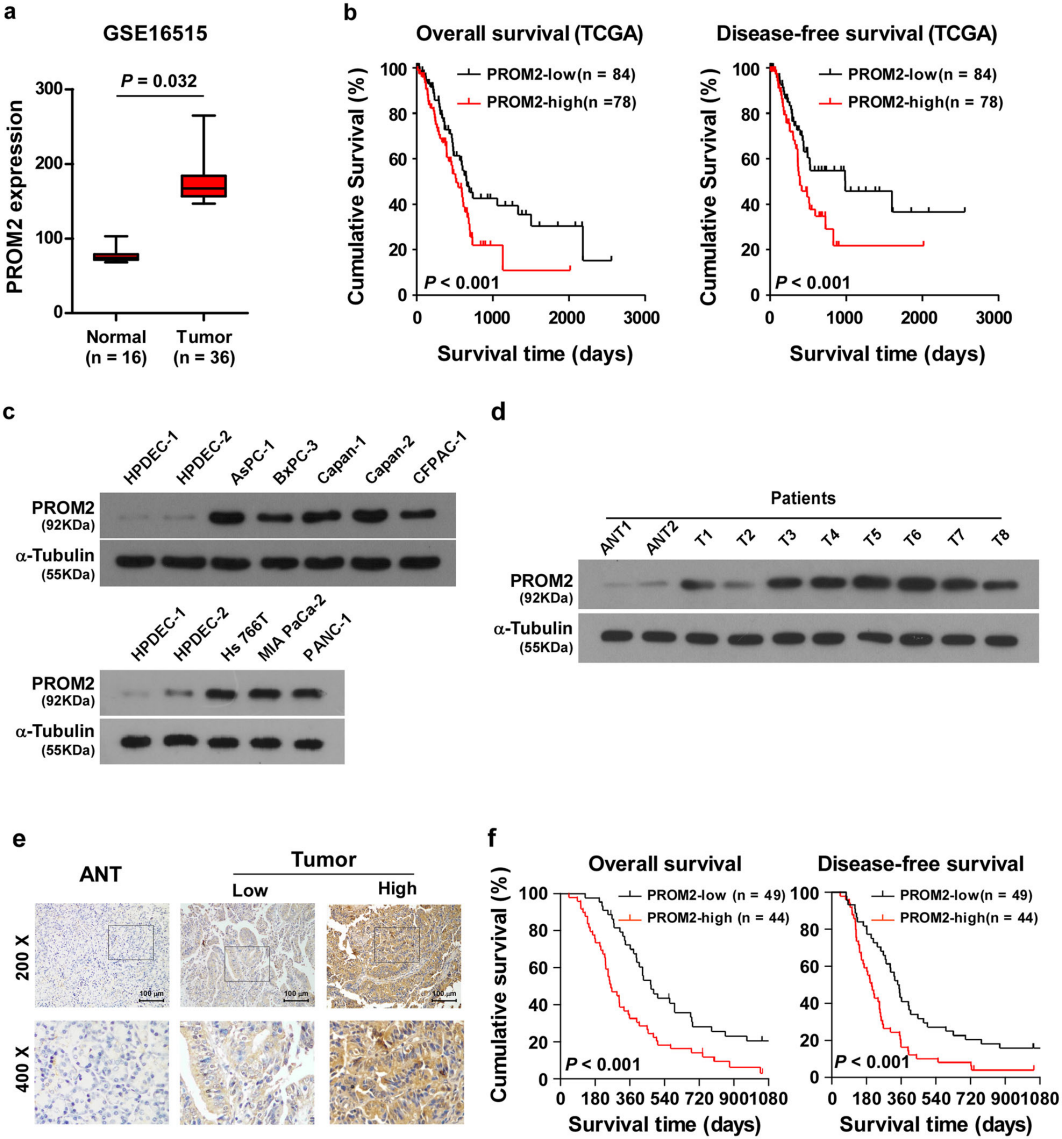
Overexpression of PROM2 is positively correlated with pancreatic cancer progression. **a** PROM2 is overexpressed in pancreatic tumor tissues versus normal tissues (NCBI/GEO/GSE16515, *P* = 0.032). **b** High expression of PROM2 is correlated with poor overall survival and disease-free survival in pancreatic cancer (*P* < 0.001, *P* < 0.001; TCGA, *n* = 162). **c** PROM2 protein expression is upregulated in all eight pancreatic cancer cell lines compared with immortal pancreatic ductal epithelial cell (HPDECs). **d** PROM2 protein is overexpressed in eight pancreatic cancer tissues (T1–T8) versus adjacent tissues of pancreatic cancer (N1, N2). **e** Representative images of adjacent tissues and tumor tissues stained with the PROM2 antibody by IHC assay. **f** Correlation between PROM2 expression and overall/disease-free survival (*P* < 0.001, *P* < 0.001; *n* = 93).

The cohort of 93 patients from Figs. 1e, f, and 7c were paraffin-embedded, archived specimens obtained from Sun Yat-Sen Memorial Hospital between 2001 and 2014, which were diagnosed histopathologically and clinically as pancreatic cancer. The Ethical approval number of this study (including pancreatic cancer and adjacent normal tissues) was [2017]-183. The protein samples of T1–T8 tumor tissues and ANT1-2 (adjacent normal tissues of T1–T2) in Fig. 1d and Supplementary Fig. S2b (Ethical approval number: [2012]-12), and T1–T10 tumor tissues in Fig. 7a (Ethical approval number: [2018]-057) were extracted from freshly collected pancreatic cancer tissues before receiving gemcitabine-based treatment. The clinical information of the patients involved in the study is shown in Supplementary Table S1. Prior donor consent was obtained from all patients. Approvals from Institutional Research Ethics Committee of Sun Yat-Sen Memorial Hospital were also obtained for this research.

### Cells

The pancreatic cancer cell lines, including AsPC-1, Bxpc-3 were grown in the RPMI-1640 Medium (Invitrogen, Carlsbad, CA), Capan-1 and CFPAC-1 were grown in the Iscove’s modified Dulbecco’s medium (Invitrogen, Carlsbad, CA), Capan-2 was grown in the McCoy’s 5a Medium Modified Medium (Invitrogen, Carlsbad, CA), Hs 667T, MIA PaCa-2, and PANC-1 were grown in the DMEM medium (Invitrogen, Carlsbad, CA) supplemented with 10% fetal bovine serum (HyClone, Logan, UT). Primary cultures of immortal human pancreatic duct epithelial cells (HPDECs) were maintained in keratinocyte serum-free medium (KSFM; Gibco, Grand Island, NY, USA) with EGF (1 ng/ml) and BPE (50 mg/ml). All cells were incubated at 37 °C in a humidified atmosphere with 5% CO_2_.

### Western blot analysis

Cell lysates were separated by 10% sodium dodecyl sulfate-polyacrylamide gel electrophoresis and then transferred to polyvinylidene fluoride membranes (Millipore, Billerica, MA, USA). The membranes were incubated with antibodies against PROM2 (1:500, Abcam, Cambridge, MA, USA), p-Akt (Ser473) (1:2000, Cell Signaling Technology, Danvers, MA, USA), Akt1 (1:1000, Cell Signaling Technology), p-BAD (Ser136) (1:500, Cell Signaling Technology), BAD (1:1000, Cell Signaling Technology), p-Caspase-9 (Ser 196) (1:500, Abcam), Caspase-9 (1:1000, Abcam), Flag (1:1000, Sigma, St Louis, MO, USA) and HA (1:500, Sigma) overnight at 4 °C, then incubated with horseradish peroxidase-conjugated secondary antibodies (Goat anti-rabbit/mouse, PIERCE, Waltham, MA, USA) for 1 h at room temperature. The blotting membranes were stripped and re-probed with an anti-α-Tubulin antibody (1:1000, Sigma).

### RNA extraction, reverse transcription (RT), and real-time PCR

Total RNA from cultured cells and freshly collected pancreatic cancer tissues was extracted using TRIzol (Life Technologies, Waltham, MA, USA) according to the manufacturer’s instructions. Reverse transcription (RT) of total mRNA was performed using a PrimeScript RT Reagent kit (TaKaRa, Kyoto, Japan) according to the manufacturer’s protocol. cDNAs were amplified and quantified in a Bio-Rad CFX qRT-PCR detection system (Applied Biosystems Inc., Foster City, CA, USA), using SYBR Green Master (ROX; Roche, Toronto, ON, Canada). Expression data were normalized to the geometric mean of housekeeping gene GAPDH to control the variability in expression levels and calculated as 2^[(Ct of gene) − (Ct of GAPDH)]^ (Ct represents the threshold cycle for each transcript).

### Immunohistochemistry (IHC) assays

Immunohistochemical analysis was performed to elucidate the protein expression in 93 human pancreatic cancer tissues. The degree of immunostaining of paraffin-embedded sections was reviewed and scored by two observers independently, jointly based on the proportion of positively stained tumor cells and the intensity of staining. The proportion of tumor cells was graded as follows: 0 (no positive tumor cells), 1 (<10% positive tumor cells), 2 (10–50% positive tumor cells), and 3 (>50% positive tumor cells). The intensity of staining was scored according to the following criteria: 0 (no staining); 1 (light yellow), 2 (yellow brown), and 3 (brown). The staining index (SI) was calculated by multiplying the scores of the proportion of positive tumor cells and the staining intensity. By using this method of assessment, we evaluated the expression of indicated proteins in pancreatic cancer and adjacent samples by determining the SI, which scores as 0, 1, 2, 3, 4, 6, and 9. The cutoff values chosen for PROM2 or p-Akt were on the basis of a measurement of heterogeneity according to the log-rank test statistical analysis of overall/disease-free survival. The optimal cutoff value of the SI scores was identified as: ≥4 defined as tumor with high expression, while ≤3 defined as low expression. The antibodies used in the IHC experiments were PROM2 (1:250, Sigma), Akt1 (1:1000, Cell Signaling Technology), and p-Akt (Ser473) (1:100, Cell Signaling Technology).

### Vectors, retroviral infection and transfection

The pSin/PROM2 with human gene PROM2 overexpression was established by sub-cloning the PCR-amplified PROM2 coding sequence into pSin vector. To silence the endogenous PROM2 and Akt1, two RNAi oligonucleotides were cloned into the pSuper-retro-puro vector to generate pSuper-retro-puro-PROM2-RNAi(s), pSuper-retro-puro-Akt1-RNAi(s), respectively. Transfection procedures of plasmids were performed by using the Lipofectamine 3000 (Life Technologies) according to the manufacturer’s instructions. Stable cell lines expressing PROM2 or PROM2 shRNAs were generated via retroviral infection using HEK293T cells and selected with 0.5 μg/ml puromycin 48 h after infection. After 10-day selections, the cell lysates prepared from the pooled population of cells using sample buffer were further fractionated on SDS-PAGE for verifying the expression of PROM2 protein level.

### Primers and oligonucleotides

The primers used for cloning PROM2-FlAG: 5′-gccGGATCCATGGACTACAAGGA CGACGATGACAA GAAGCACACACTGGCTCTGCTGGC-3′ and 5′-gccGAATTCCTA CAGCTTCAGGGAG GTAACCCGG-3′; Cloning HA-Akt: 5′-gccGGATCCATGTACCC ATACGATGTTCCAG ATTACGCTAGCGACGTGGCTATTGTGAAGG-3′ and 5′-gccGAATTCTCAGGCCGTG CCGCTGGCCGAGTA-3′. PROM2 PCR primer: 5′-AGGTCCAGGCTCTGTGTGTC-3′ and 5′-GCTCAACGACTCCTACGACC-3′. For depletion of PROM2 and Akt1siRNAs was synthesized and purified by RIBOBIO Company (Guangzhou, China).

### Cell clonogenic survival assay

The indicated Cells were plated in 24-well plates (8 × 10^2^ cells per plate) and cultured for 10 days. The colonies were stained with 1% crystal violet for 30 min followed by fixation with 10% formaldehyde for 10 min. The number of colonies (defined as cell clusters composed of more than 50 cells) was quantified by Analysis software (Olympus Biosystems).

### MTT cell viability assay

The indicated cells (2 × 10^4^ cells per plate) in 48-well plates were transfected appropriate siRNAs. After 48 h, each cell was further treated for 24 h with gemcitabine (50 nM), then stained with 100 μl sterile 3-(4,5-Dimethyl-2-thiazolyl)-2,5-diphenyl-2H-tetrazolium bromide (MTT) dye (0.5 mg/ml, Sigma) for 4 h at 37 °C, after removal of the culture medium and addition of 150 μl of dimethyl sulphoxide (DMSO) (Sigma). The absorbance was measured at 570 nm, which regarding 655 nm as the reference wavelength. Dose–response curves were plotted on a semilog scale according to the percentage of the control cell number that was obtained from the sample with no drug exposure. The IC_50_ values were calculated using the GraphPad Prism® 5 software (Version 5.01, GraphPad Software, Inc., USA).

### Annexin-V assay

The ApopNexinTM FITC Apoptosis Detection Kit (Millipore) was used for the quantification of apoptotic cells in indicated cells, followed the manufacturer’s instruction. Briefly, indicated treated cells were firstly washed with PBS twice and then responded with the Annexin-V binding solution, subsequently added 150 μl of the Annexin-V antibody in Binding Buffer and incubated for 15 min. Afterward, addition of 1.5 μl of PI at the concentration as 1 mg/ml and a further incubation for 5 min were processed. After washing with the Annexin-V Binding Buffer, positive Annexin-V staining was visualized under a Flow Cytometer equipped with two panels for fluorescein isothiocyanate (excitation: 490 nm, emission: 525 nm), and PI staining was assessed with the filter for Texas red (excitation: 570 nm, emission: 610 nm).

### Xenografted tumor models

The indicated cells (1 × 10^6^) were subcutaneously injected into the space underneath the skin of Balb/c nude mice. Tumor growth was monitored by measuring the tumor luminescence signals by utilizing the Living image system. When the luminescence signal reached 2 × 10^7^ p/s/cm^2^/sr, mice were intravenously treated with vehicle (control) or gemcitabine (80 mg/kg body weight, twice every week), gemcitabine plus control or gemcitabine plus MK-2206 (120 mg/kg body weight, three times per week) for up to 6 weeks. The luminescence signal was recorded every week. At the end of treatment, the mice were sacrificed and the tumors were removed, excised and weighed. The tumor volume was monitored with length (*L*) and width (*W*), and calculated using the equation (*L* × *W*^2^)/2.

### Immunofluorescence (IF) staining

The immunofluorescent staining was carried out in paraffin-embedded tumor tissues formed by the indicated cells. The staining assays were processed by using the antibody cleaved-Caspase-3 (1:200, Cell Signaling Technology) or staining with TUNEL (In Situ Cell Death Detection Kit, TMR red, Roche Applied Science, Penzberg, Germany). The images were captured using the AxioVision Rel.4.6 computerized image analysis system (Carl Zeiss).

### Immunoprecipitation assay

Cell lysates were prepared from 5 × 10^7^ AsPC-1 transfected with indicated plasmids using lysis buffer (150 mM NaCl, 10 mM HEPES, pH 7.4, 1% NP-40). Then the lysates were incubated overnight with FLAG/HA affinity agarose (Sigma-Aldrich) at 4 °C. Beads containing affinity-bound proteins were washed six times by immunoprecipitation wash buffer (150 mM NaCl, 10 mM HEPES, Ph 7.4, 0.1% NP-40), followed by elution twice with 200 μl of 1 M glycine (pH 3.0). Subsequently, the eluates were pooled and concentrated in a 10-kDa MW cut-off filter unit (Millipore) up to a volume of 30 μl. After adding 10 μl of 4 × sample buffer and denaturation, proteins were separated on SDS polyacrylamide gels and performed as western blotting assays.

### Far-western blotting analysis

The far-western blotting assay was performed according to a previous report^35^. In brief, a plasmid encoding Flag-tagged PROM2 was transfected into AsPC-1 cells and immunoprecipitated by Flag-tag affinity gel (Sigma-Aldrich, St. Louis, MO) and resolved by SDS-PAGE. The proteins were transferred into PVDF membrane and blocked in 10% skimmed milk for 1 h at 4 °C. Recombinant GST-AKT protein was added at 5 μg/ml and incubated at 4 °C for 18 h. After 6 times with TBST washes, the membrane was subjected to western blotting analysis using indicated antibody.

### Statistical analysis

Student’s two-tailed *t* test was performed in statistical comparisons between two sets of data. Bivariate correlations between different study variables were calculated by Spearman’s rank correlation coefficients. Survival curves were plotted by the Kaplan–Meier method and compared via the log-rank test. Univariate and multivariate Cox regression analyses were used to analyze the significance of various variables for survival. All statistical analyses were performed using the SPSS 11.0 statistical software package. Data represent mean ± SD. *P* values of <0.05 were considered statistically significant.

## Results

### Overexpression of PROM2 is positively correlated with pancreatic cancer progression

According to the public dataset NCBI/GEO/GSE16515, PROM2 is upregulated in pancreatic cancer tissues compared with normal pancreatic tissues (*P* = 0.032; *n* = 52, Fig. 1a). We also found that higher expression of PROM2 predicted shorter overall survival and disease-free survival in the Cancer Genome Atlas (TCGA) dataset (*P* < 0.001; *P* < 0.001; *n* = 162, Fig. 1b). Consistently, both the mRNA and protein expression level of PROM2 were markedly increased in pancreatic cancer cell lines compared with immortal pancreatic ductal epithelial cell (HPDECs) (Fig. 1c and Supplementary Fig. S1a). Importantly, PROM2 was significantly upregulated in eight freshly collected pancreatic cancer tissues before gemcitabine-based treatment compared to two adjacent pancreatic tissues N1–N2 (Fig. 1d and Supplementary Fig. S1b). These findings suggest PROM2 is ubiquitously upregulated in pancreatic cancer.

Immunohistochemistry (IHC) assays showed PROM2 was overexpressed in clinical pancreatic cancer tissues comparison to adjacent pancreatic tissues (Fig. 1e), which led to poor overall survival and disease-free survival in the same cohort of cancer samples (*P* < 0.001; *P* < 0.001; *n* = 93, Fig. 1f). Statistical analysis confirmed that the expression of PROM2 was significantly correlated with clinical stages in patients with pancreatic cancer, and also indicated lower overall survival and disease-free survival rates (Supplementary Tables S1–S2). Collectively, these data demonstrate PROM2 overexpression is in a close relationship with pancreatic cancer progression, and could serve as an independent prognostic factor.

### PROM2 upregulation promotes gemcitabine chemoresistance in pancreatic cancer

**Fig. 2:**
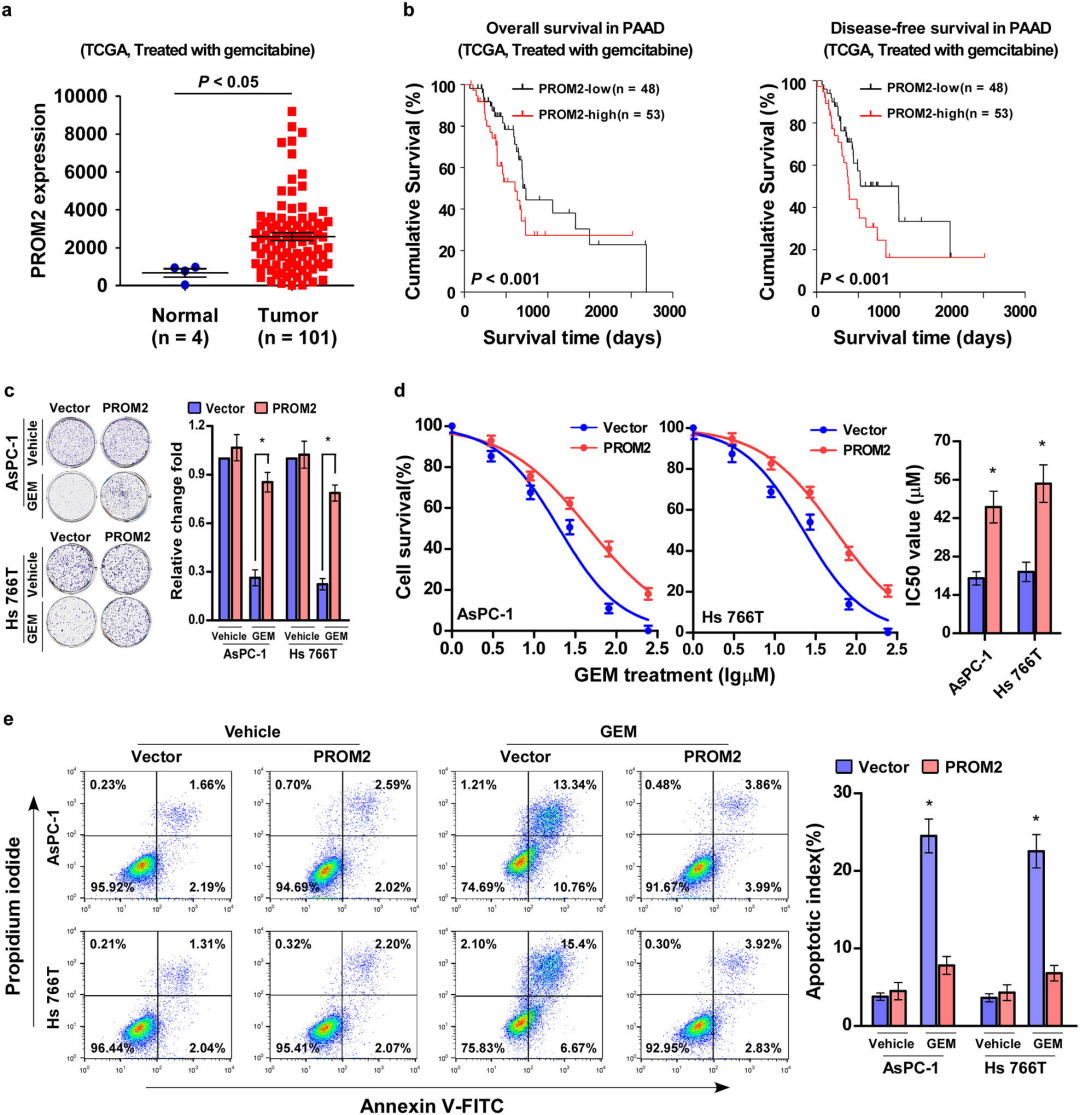
PROM2 upregulation promotes gemcitabine chemoresistance in pancreatic cancer. **a** The expression level of PROM2 in pancreatic cancer patients treated with gemcitabine. **b** High expression of PROM2 in pancreatic cancer patients treated with gemcitabine indicates poor overall and disease-free survival (*P* < 0.001, *P* < 0.001; TCGA, *n* = 101). **c** Representative images (left) and quantification (right) of colonies generated with the indicated cells treated with vehicle or gemcitabine (10 μM). The numbers of clone formation of AsPC-1/vector or Hs 766T/vector has been set for control at 1 (mean ± SD, *n* = 3; *P < 0.05). **d** MTT cell viability assay (left) at different concentrations and IC_50_ value of Gemcitabine (right, 10 μM) in the indicated cells (mean ± SD, *n* = 3; *P < 0.05). **e** FACS analysis of Annexin-V and PI staining (left) and quantification (right) of indicated cells treated with Gemcitabine (10 μM) (mean ± SD, *n* = 3; *P < 0.05).

To further investigate the regulatory role of PROM2 in tumor progression, pancreatic cancer patients who were treated with gemcitabine were selected for survival analysis. PROM2 overexpression resulted in much shorter overall survival and disease-free survival times in pancreatic cancer patients who were treated with gemcitabine chemotherapy (*P* < 0.001; *P* < 0.001; *n* = 81, Fig. 2a, b, Supplementary Table S3). These data suggest PROM2 is linked to gemcitabine chemoresistance.

To test the hypothesis, pancreatic cancer cell lines AsPC-1 and Hs 766T stably expressing PROM2 were constructed (Supplementary Fig. S2a). PROM2 upregulation dramatically increased the colony-forming ability of pancreatic cancer cell lines AsPC-1 and Hs 766T when treated with gemcitabine, and did not show obvious alterations when treated with vehicle (Fig. 2c). In addition, the half maximal inhibitory concentration (IC_50_) values of gemcitabine were greatly increased in PROM2 overexpressing cells (Fig. 2d). FACS analysis of Annexin-V and PI staining-indicated lower apoptotic rates in PROM2 overexpressing cells treated with gemcitabine, and showed no significant difference when treated with vehicle (Fig. 2e). Consistently, the colony formation and Annexin-V assays revealed that overexpression of PRMO2 significantly increased the capability of CFPAC-1 cell on gemcitabine resistance (Supplementary Fig. S2b, c). These data confirm PROM2 plays a pivotal role in gemcitabine chemoresistance in pancreatic cancer.

### Silencing of PROM2 induces gemcitabine chemosensitivity of pancreatic cancer cells

**Fig. 3:**
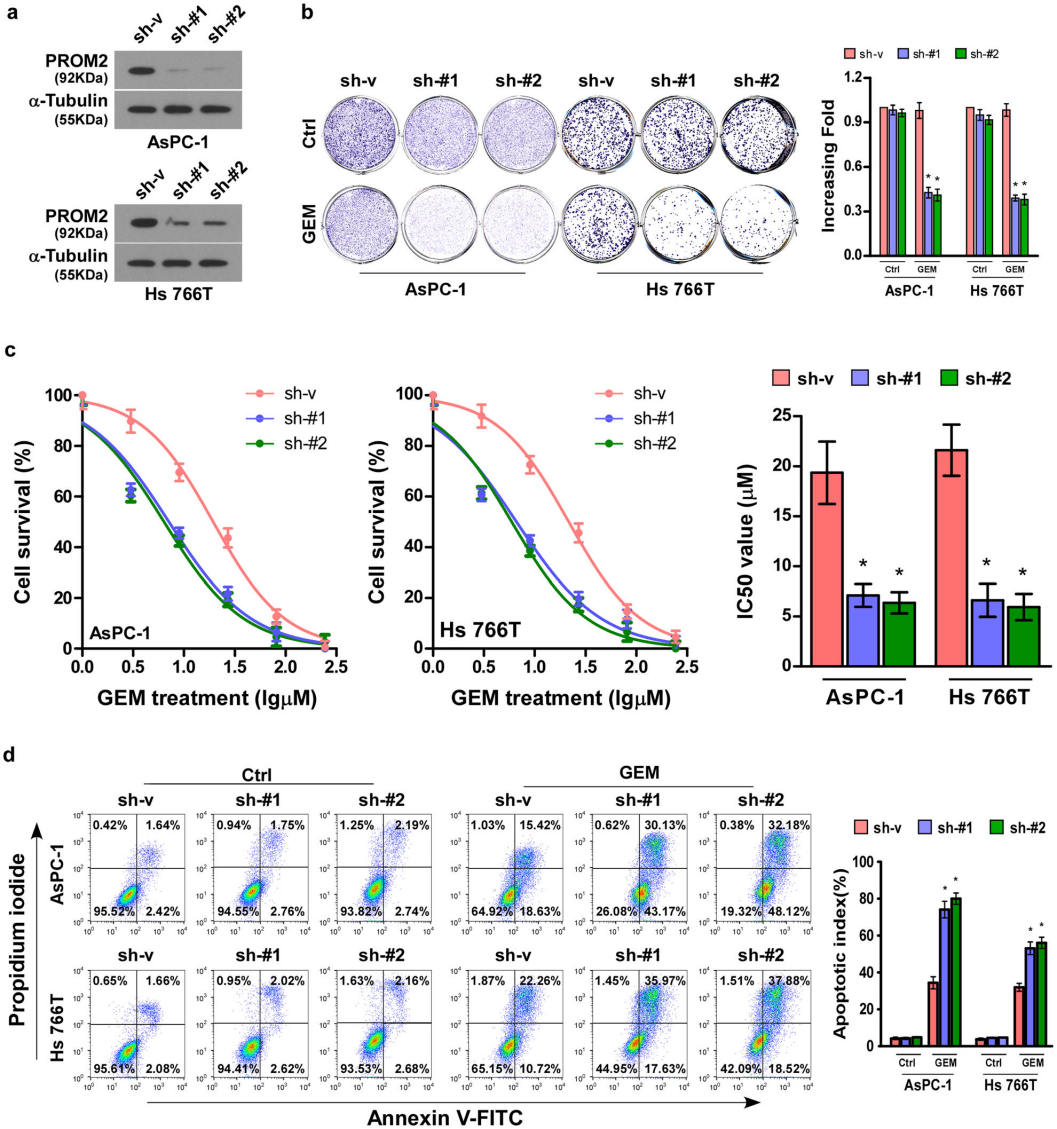
Silencing of PROM2 induces gemcitabine chemosensitivity in pancreatic cancer cells. **a** Western blot of PROM2 protein expression in indicated cells. **b** Representative images (left) and quantification data (right) of colony numbers in the indicated cells treated with vehicle or gemcitabine (10 μM) (mean ± SD, *n* = 3; *P < 0.05). **c** MTT cell viability assay and IC_50_ value of gemcitabine (10 μM) in the indicated cells (mean ± SD, *n* = 3; *P < 0.05). **d** FACS analysis of Annexin-V and PI staining (left) and quantification (right) of indicated cells treated with Gemcitabine (10 μM) (mean ± SD, *n* = 3; *P < 0.05).

To further evaluate the biological effect of PROM2 in the development of pancreatic cancer cell chemoresistance, PROM2-silenced stable cell lines (AsPC-1 and Hs 766T) were produced (Fig. 3a and Supplementary Fig. S2d). Downregulation of PROM2 did not change the colony formation ability of AsPC-1 and Hs 766T pancreatic cancer cell lines without gemcitabine treatment. However, both PROM2-silenced AsPC-1 and Hs 766T cells presented weaker colony-forming capacity under the pressure of gemcitabine treatment (Fig. 3b). Similarly, downregulation of PROM2 lowered the IC_50_ value of gemcitabine in pancreatic cancer cell lines (Fig. 3c). In addition, the apoptotic proportion of PROM2-knockdown cells after gemcitabine treatment was higher than in vector control cells, but showed no difference compared with vehicle treatment (Fig. 3d). In conclusion, PROM2 knockdown augments the chemosensitivity of pancreatic cancer cells to gemcitabine treatment.

### PROM2 enhances gemcitabine chemoresistance in pancreatic cancer in vivo

**Fig. 4:**
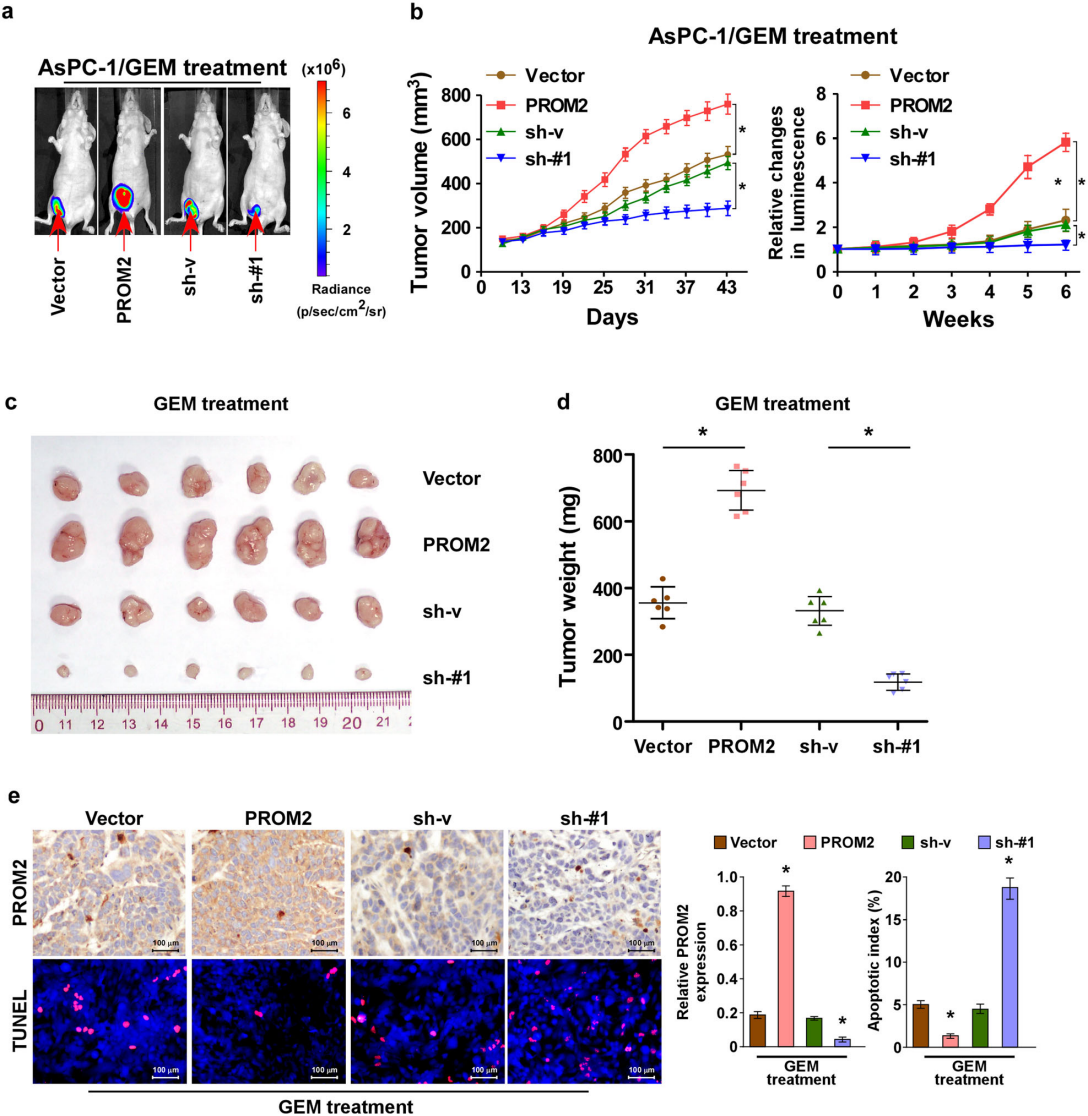
PROM2 enhances gemcitabine chemoresistance in pancreatic cancer in vivo. **a** Luminescence signal of xenografted tumors formed by indicated cells in mice after gemcitabine treatment. **b** The changes of tumor volume and luminescence with gemcitabine treatment in indicated cells (mean value is plotted). **c** Representative images of tumor mass removed from mice with xenografted tumors. **d** Tumor weight (mg) of the collected tumor mass removed from mice xenografted with indicated cancer cells (*P < 0.05). **e** Representative images of PROM2 and TUNEL immunostaining in indicated tumor tissues; the quantification of results is shown on the right panel (*P < 0.05), the PROM2 expression of vector group has been set as 1.

As we proved PROM2 promotes chemoresistance in vitro, next we tested the role of PROM2 in vivo. AsPC-1 cells (with different levels of PROM2 expression) were infected with luciferase and subcutaneously injected into the back of Balb/c nude mice (Fig. 4a). When the luminescence signal reached 2 × 10^7^ p/s/cm^2^/sr, the mice were administered intraperitoneally with gemcitabine. Weekly measurements of the luminescence signal showed that overexpression of PROM2 accelerated the growth rate of the tumor compared with vector control cells, while silencing of PROM2 inhibited tumor growth (Fig. 4a, b).

The mice were sacrificed after 7 weeks of gemcitabine treatment and subcutaneous tumors were removed. Upregulation of PROM2 increased (while downregulation of PROM2 decreased) both the tumor volume and weight in vivo with gemcitabine treatment (Fig. 4c, d). The expression of PROM2 was confirmed with IHC analysis (Fig. 4e). Congruously, PROM2 overexpression reduced the proportion of terminal deoxynucleotidyl transferase dUTP nick end labeling (TUNEL) cells, while PROM2 knockdown displayed the opposite results (Fig. 4e). Overall, our in vivo data indicate PROM2 promotes chemoresistance in pancreatic cancer.

### PROM2 activates the Akt signaling pathway

**Fig. 5:**
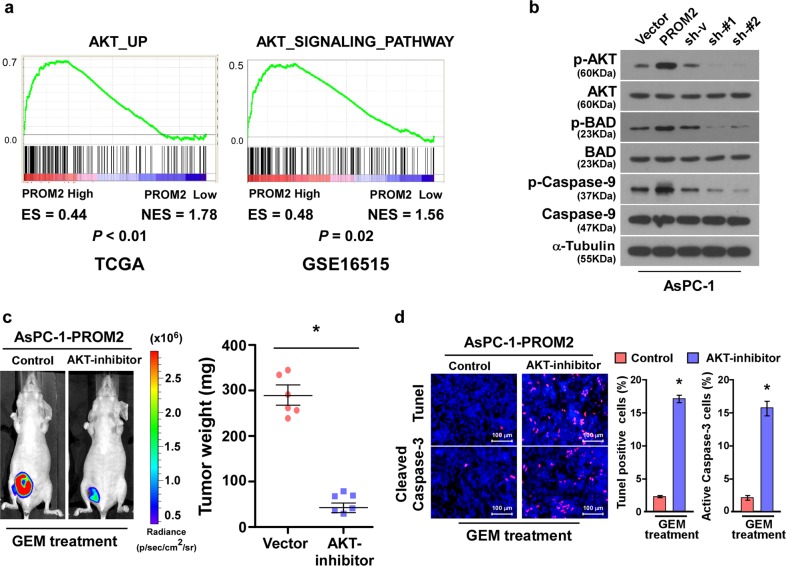
PROM2 activates the Akt signaling pathway. **a** GSEA analysis showing the positive correlation between PROM2 expression and gene sets relevant to the Akt signaling pathway. **b** Immunoblotting assays demonstrating the expression level of the indicated proteins in AsPC-1 cells with different expression level of PROM2. **c** Luminescence signaling (left) and tumor weight (right) of the tumors formed by AsPC-1/PROM2 after treatment with vehicle or the Akt inhibitor (MK-2206) (*P < 0.05). **d** Representative images (left) and quantification (right) of TUNEL and active caspase-3 in tumors with the indicated treatment (*P < 0.05).

Constitutive activation of the Akt signaling pathway occurs in various cancer types and confers chemoresistance^13,36–38^. To determine whether PROM2 promotes chemoresistance via modulation of the Akt pathway, GSEA enrichments were executed and revealed PROM2 expression positively correlated with the Akt signaling pathway related gene signature (Fig. 5a). Similar observations were also obtained with immunoblotting, which demonstrated PROM2 upregulation promoted (while PROM2 knockdown inhibited) the phosphorylation of Akt (Fig. 5b). The main targets of Akt (i.e., BAD and Caspase-9) were also phosphorylated as PROM2 was overexpressed, and phosphorylation levels of BAD and Caspase-9 were dramatically inhibited in PROM2-silenced cells (Fig. 5b).

To confirm whether PROM2-induced chemoresistance was dependent upon the Akt pathway, tumor-bearing mice injected with PROM2-upregulated cells were treated with combination of gemcitabine and vehicle, or gemcitabine and an Akt inhibitor MK-2206. Strikingly, the luminescence signal and tumor weight were significantly decreased in mice treated with gemcitabine and the Akt inhibitor and indicated slightly changes treated with Akt inhibitor alone and AsPC-1/vector cells (Fig. 5c and Supplementary Fig. S3b, c). Consistently, the proportion of TUNEL and active Caspase-3 positive cells were increased (Fig. 5d), suggesting the Akt inhibitor renders pancreatic cancer cell sensitive to gemcitabine treatment.

### PROM2 augments gemcitabine chemoresistance by binding to Akt

**Fig. 6:**
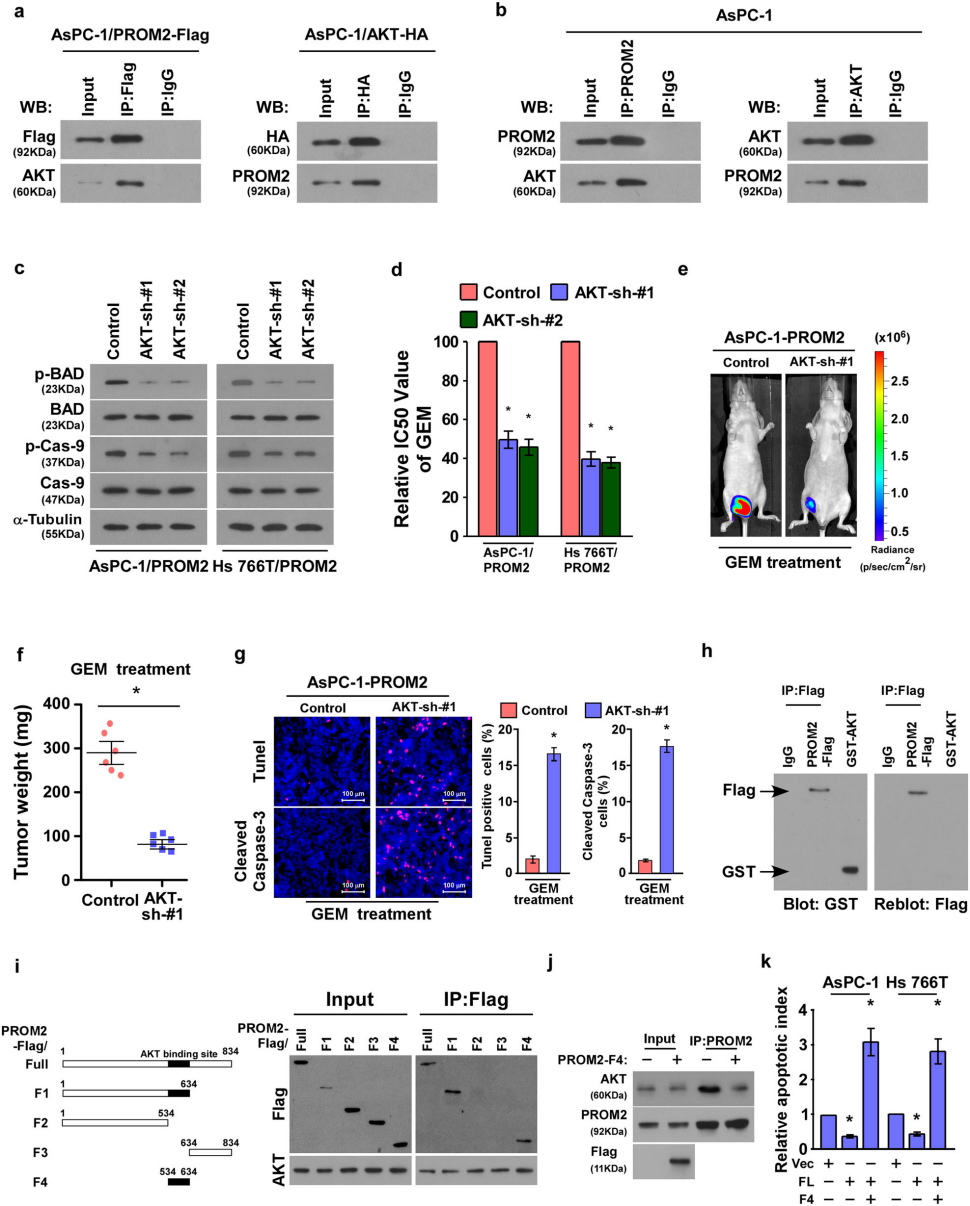
PROM2 augments gemcitabine chemoresistance by binding to Akt. **a** Immunoprecipitation assays showing PROM2 interacts with Akt. **b** PROM2 binds to Akt endogenously. **c** Protein expressions of p-BAD and p-Caspase-9 are inhibited when silencing Akt. **d** IC_50_ value of gemcitabine in the indicated cells after Akt knockdown (*P < 0.05). **e** Luminescence signaling of tumors formed by AsPC-1/PROM2 cells after treatment with control or Akt shRNA (*P < 0.05). **f** Tumor weight of tumors formed by AsPC-1/PROM2 cells after treatment with control or Akt shRNA (*P < 0.05). **g** Representative images of the immunostaining in tumors tissues (*P < 0.05). **h** Far-western blotting indicated that Flag-PROM2 interacted with recombinant GST-AKT directly. **i** Schematic illustration of full length PROM2 and truncations (left) and co-IP assays revealed AKT bound with FL, F1, and F4 fragments, but not with F2 and F3 fragments (right). **j** Immunoprecipitation assays illustrating the inhibitory effect of PROM2-F4 on the PROM2-AKT interaction. **k** Quantification of Annexin-V apoptotic cells in indicated cells treated with Gemcitabine (10 μM) (mean ± SD, *n* = 3; *P < 0.05).

Akt, also known as protein kinase B (PKB), is the prominent component in the Akt signaling pathway. Akt resides in the cytoplasm in an inactive conformation and translocates to the plasma membrane when the pathway is stimulated^15,16^. Reciprocal co-immunoprecipitation and western blotting assays were performed using AsPC-1 cells, and the assay demonstrated that PROM2 interacts with AKT (Fig. 6a). The interaction between endogenous PROM2 and AKT was also verified in AsPC-1 cells (Fig. 6b). Knockdown of AKT in AsPC-1 and Hs 766T cells overexpressing PROM2 (Supplementary Fig. S3) inhibited the downstream effectors of the Akt signaling pathway (i.e., phosphorylated AKT, p-BAD, and p-Caspase-9) (Fig. 6c). Importantly, the IC_50_ value of gemcitabine in AsPC-1/PROM2 cells were greatly decreased with AKT knockdown (Fig. 6d), as well as the tumor luminescence and tumor weight (Fig. 6e, f). Meanwhile, the proportion of TUNEL and active Caspase-3 positive cells were increased with AKT knockdown (Fig. 6g), which implies that PROM2 facilitates gemcitabine chemoresistance through binding to AKT.

Importantly, far-western blotting showed that PROM2 directly interacted with AKT (Fig. 6h). To determine the region of PROM2 responsible for AKT binding, several PROM2 truncations were established (Fig. 6i). Co-IP assays with anti-Flag antibody demonstrated that AKT interacted with PROM2-FL (full length), F1 (aa 1–634) and F4 (aa 534–634), but could not bind with F2 (aa 1–534) and F3 (aa 634–834) fragments, suggesting that a region between 534 and 634aa was required for PROM2-AKT interaction (Fig. 6i). Furthermore, the inhibitory effect of PROM2-F4 (aa 534–634) on the PROM2-AKT interaction has been verified using co-IP assay (Fig. 6j). Consistently, overexpressing PROM2-F4 in AsPC-1 and Hs 7667 cells significantly abrogated the inhibitory effect of PROM2 on gemcitabine treatment, as indicated by increased apoptotic cells and decreased colony formation (Fig. 6k and Supplementary Fig. S3d). These results suggest that PROM2-AKT binding is required for PROM2-mediated gemcitabine resistance in pancreatic cancer cells.

### Clinical relevance of PROM2/Akt signaling pathway in pancreatic cancer

**Fig. 7:**
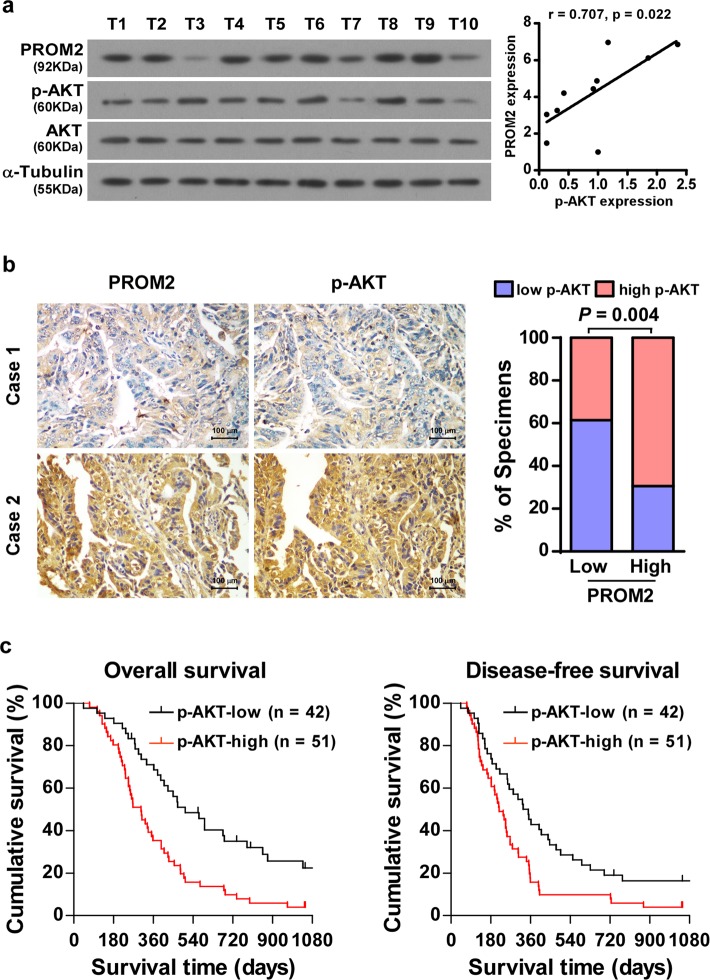
Clinical relevance of PROM2-induced activation of Akt signaling in pancreatic cancer. **a** PROM2 is positively correlated with p-Akt in 10 freshly collected pancreatic cancer tissues (*r* = 0.707, *P* = 0.022). **b** Immunohistochemistry assays revealing PROM2 is positively correlated with p-Akt in 93 paraffin-embedded pancreatic cancer samples (*P* = 0.004). **c** P-Akt expression predicted poor overall (left) and disease-free (right) survival in pancreatic cancer samples (*P* < 0.001, *P* < 0.001).

To better define whether PROM2 is clinically correlated with the Akt signaling pathway, we examined the expression levels of PROM2 and AKT in 10 freshly collected pancreatic cancer tissues. The expression of PROM2 and p-AKT proteins was positively correlated in pancreatic cancer tissues (*P* = 0.022, *r* = 0.688, Fig. 7a). The same relationship was observed in clinical pancreatic cancer tissues using IHC (*P* = 0.004, Fig. 7b). Most importantly, the high levels of p-AKT led to poorer overall survival and times in patients with pancreatic cancer (Fig. 7c). These results indicate the vital clinical role of PROM2-induced activation of the Akt signaling pathway disease-free survival in pancreatic cancer.

## Discussion

PDAC patients are mostly diagnosed at locally advanced or metastatic stages, in which limited response to current treatments results in an extremely poor prognosis^39^. Therefore, to unveil the early predictive marker for existing therapies are urgently needed. Our data suggest PROM2 is dramatically and ubiquitously upregulated in cancer tissues and cell lines, and leads to shorter overall and disease-free survival time. In addition, overexpression of PROM2 in pancreatic cancer cells augments gemcitabine chemoresistance, both in vivo and in vitro. The molecular modulatory mechanism of PROM2 in chemoresistance has also been deciphered: PROM2 could directly binds with Akt and strengthens the signaling transduction of the Akt signaling pathway. Finally, we showed combining gemcitabine with an Akt pathway inhibitor (MK-2206) or Akt shRNA restores the sensitivity of pancreatic cancer cells to gemcitabine chemotherapy and improves the survival status of experimental mice.

The incorporation of chemotherapy drug gemcitabine into the DNA creates an irreparable error, which inhibits further DNA synthesis, induces cell apoptosis^40,41^. Resistance is a major cause of treatment failure in chemotherapy among cancer patients^42,43^. There are several signaling pathways, including Akt pathway, MAPK, Bcl-2, and MMP13 pathways, involved with gemcitabine chemoresistance in pancreatic cancer^13,44^. Though previous reports have suggested that MUC1, MUC4, and DNA-PKcs might enhance pancreatic cancer chemoresistance^45–47^, the underlying mechanism remains largely unknown. Our data suggest that PROM2 induces gemcitabine resistance via hyper-activation of the Akt signaling pathway, which inactivates BAD, Caspase-9, and eventually hinders the gemcitabine-induced apoptotic cascade. We also found overexpression of phosphorylated Akt was associated with a low overall survival rate and high relapse frequency, which also resulted in gemcitabine chemoresistance in patients with pancreatic cancer^13,44^. This result confirms the significant role of the Akt pathway in gemcitabine resistance and highlights the molecular regulatory mechanism of Akt signaling in pancreatic cancer. Since the aberrant activation of the Akt signaling pathway confers resistance to traditional chemotherapy, search for therapeutic strategies to complement chemotherapy regimens has advanced to include Akt inhibitors^19,48^. Preclinical study has further underscored the potential value of Akt inhibitors in multiple types of cancer, including head and neck cancer (NCT01349933), pancreatic cancer (NCT01783171), and ovarian cancer (NCT01283035). Among the various kinds of Akt inhibitors, the allosteric inhibitor MK-2206 is the most common compound used in cancer treatments^11,18,48^. The insurmountable obstacle to the usage of Akt inhibitors lies in the selection of the specific subtypes of cancer patients. Mutations and epigenetic downregulation of PTEN (phosphatase and tensin homologue) have been regarded as crucial for Akt signaling activation and PDAC chemoresistance^49,50^. Moreover, HEATR1 negatively regulates Akt and sensitize pancreatic cancer cells to chemotherapy^13^. However, the clinical outcomes of PTEN or HEATR1 in PDAC has to be further tested. Our data indicate PROM2 enhanced gemcitabine chemoresistance in vitro and in vivo, which implies that PROM2 could serve as a biomarker for co-therapy of gemcitabine and Akt inhibitors in pancreatic cancer treatment.

The existing biomarkers (such as CA19-9) show inadequate potential for early predictor for treatment response due to low sensitivity and specificity^51^, which drew great attention to the identification of prognostic biomarker for PADC. While PROM1 is broadly reported and recognized as a marker for cancer stem cells^27–29^, few studies have investigated the biological function of PROM2. PROM2 has been implicated as a marker of distal tubules and collecting ducts in the kidney^30^ and may decrease caveolae formation in fibroblasts and Chinese hamster ovary cells^32^. PROM2 has also been demonstrated to be upregulated in subtypes of lung cancer and chromophobe renal cell carcinoma^31,33^. Here we showed that PROM2 is overexpressed in pancreatic cancer and positively correlated with overall and disease-free survival of PDAC patients. In addition, the overexpression of PROM2 indicates stronger resistance to gemcitabine and causes higher relapse rates in patients with pancreatic cancer. Mechanistically we showed PROM2 could interact with Akt directly, promotes its phosphorylation and signaling transduction, which subsequently inhibits the apoptotic cascade and leads to gemcitabine chemoresistance. Therefore, our results offer new insight into both the biological function and molecular regulation of PROM2, rendering PROM2 a prominent oncogene involved in the chemoresistance of pancreatic cancer.

In summary, our findings not only uncover a novel regulatory approach underlying gemcitabine resistance and aberrant activation of the Akt signaling pathway, but also offer new insight into tailoring the specific combination regimen for patients with pancreatic cancer.

## Supplementary information

Supplemental material
