# Supplementary material for: PROM2 promotes gemcitabine chemoresistance via activating the Akt signaling pathway in pancreatic cancer
Source: Exp Mol Med. 2020 Mar 2;52(3):409–22. doi: 10.1038/s12276-020-0390-4 (PMC7156657; doi:10.1038/s12276-020-0390-4)
Supplement: Supplementary file 1 — Supplemental material [file 12276_2020_390_MOESM1_ESM.doc]

**Supplementary Table S1. The relationship between PROM2 and clinical pathological characteristics in 93 patients with pancreatic cancer.**

|  |  | PROM2 expression | |  |
| --- | --- | --- | --- | --- |
| Parameters | Number of cases | High | Low | *P* values |
| Sex |  |  |  |  |
| Male | 54 | 29 | 25 | 0.8361 |
| Female | 39 | 20 | 19 |
| Age (years) |  |  |  |  |
| <60 | 41 | 19 | 22 | 0.302 |
| ≥60 | 52 | 30 | 22 |
| Location |  |  |  |  |
| Head/neck | 66 | 37 | 29 | 0.364 |
| Body/tail | 27 | 12 | 15 |
| Tumor size (cm) |  |  |  |  |
| <2 | 30 | 1 | 29 | < 0.001 |
| ≥2 | 63 | 48 | 15 |
| Histologic grade |  |  |  |  |
| High/moderate | 32 | 12 | 20 | 0.387 |
| Poor | 61 | 29 | 32 |
| Types of surgery |  |  |  |  |
| Whipple | 73 | 35 | 38 | 0.333 |
| Distal Pancreatectomy | 11 | 7 | 4 |
| Total Pancreatectomy | 2 | 2 | 0 |
| Others | 7 | 5 | 2 |
| Pathological stage |  |  |  |  |
| I | 16 | 4 | 12 | 0.026 |
| II - IV | 77 | 45 | 32 |
| Lymph node metastasis |  |  |  |  |
| Yes | 60 | 35 | 25 | 0.193 |
| No | 33 | 14 | 19 |
| Local and distant recurrence ^1^ |  |  |  |  |
| Negative | 8 | 1 | 7 | 0.017 |
| Positive | 85 | 48 | 37 |
| Status ^1^ |  |  |  |  |
| Dead | 80 | 46 | 34 | 0.021 |
| Alive | 13 | 3 | 10 |
| IHC status of p-AKT  Low  High | 42  51 | 15  34 | 27  17 | 0.004 |

^1^ 3 years after surgical resection

**Supplementary Table S2. Univariate and multivariate analysis of factors associated with overall survival in 93** pancreatic cancer patients.

| Characteristics | Univariate analysis | | Multivariate analysis | |
| --- | --- | --- | --- | --- |
|  | HR (95% CI) | *P* values | HR (95% CI) | *P* values |
| Sex | 0.873 (0.560-1.361) | 0.550 | 0.682  (0.429-1.082) | 0.104 |
| Age (years) | 1.063  (0.682-1.657) | 0.787 | 1.326  (0.828-2.122) | 0.240 |
| Location | 2.789  (1.611-4.829) | < 0.001* | 2.467  (1.364-4.42) | 0.003* |
| Tumor size (cm) | 2.542  (1.421-4.550) | 0.002* | 1.572  (0.670-3.689) | 0.298 |
| Histologic grade | 1.090  (0.681-1.746) | 0.719 | 0.921  (0.553-1.532) | 0.750 |
| Pathological stage | 3.371 (1.672-6.795) | 0.001* | 2.273  (0.689-7.498) | 0.007* |
| Lymph node metastasis | 1.506  (0.935-2.425) | 0.092 | 0.564  (0.314-1.015) | 0.056 |
| PROM2 | 2.188 (1.392-3.439) | 0.001* | 2.155 (1.316-3.531) | 0.002* |

HR, hazard ratio; CI, confidence interval.

**Supplementary Table S3. The clinical pathological characteristics in 101 patients with Pancreatic adenocarcinoma (TCGA).**

|  |  | PROM2 expression | |
| --- | --- | --- | --- |
| Parameters | Number of cases | High | Low |
| Sex |  |  |  |
| Male | 51 | 30 | 21 |
| Female | 50 | 23 | 27 |
| Age (years) |  |  |  |
| <60 | 34 | 22 | 12 |
| ≥60 | 67 | 31 | 36 |
| Location |  |  |  |
| Head | 82 | 41 | 41 |
| Body | 5 | 4 | 1 |
| Tail | 7 | 5 | 2 |
| Others | 7 | 3 | 4 |
| Histologic grade |  |  |  |
| High/moderate | 70 | 34 | 36 |
| Poor | 31 | 19 | 12 |
| Types of surgery |  |  |  |
| Whipple | 83 | 42 | 41 |
| Distal Pancreatectomy | 10 | 6 | 4 |
| Total Pancreatectomy | 1 | 1 | 0 |
| Others | 7 | 4 | 3 |
| Pathological stage |  |  |  |
| I | 5 | 4 | 1 |
| II - IV | 96 | 49 | 47 |
| Lymph node metastasis |  |  |  |
| Yes | 78 | 38 | 40 |
| No | 23 | 15 | 8 |
| Local and distant recurrence |  |  |  |
| Negative | 50 | 27 | 23 |
| Positive | 51 | 26 | 25 |
| Status |  |  |  |
| Dead | 35 | 17 | 18 |
| Alive | 66 | 36 | 30 |


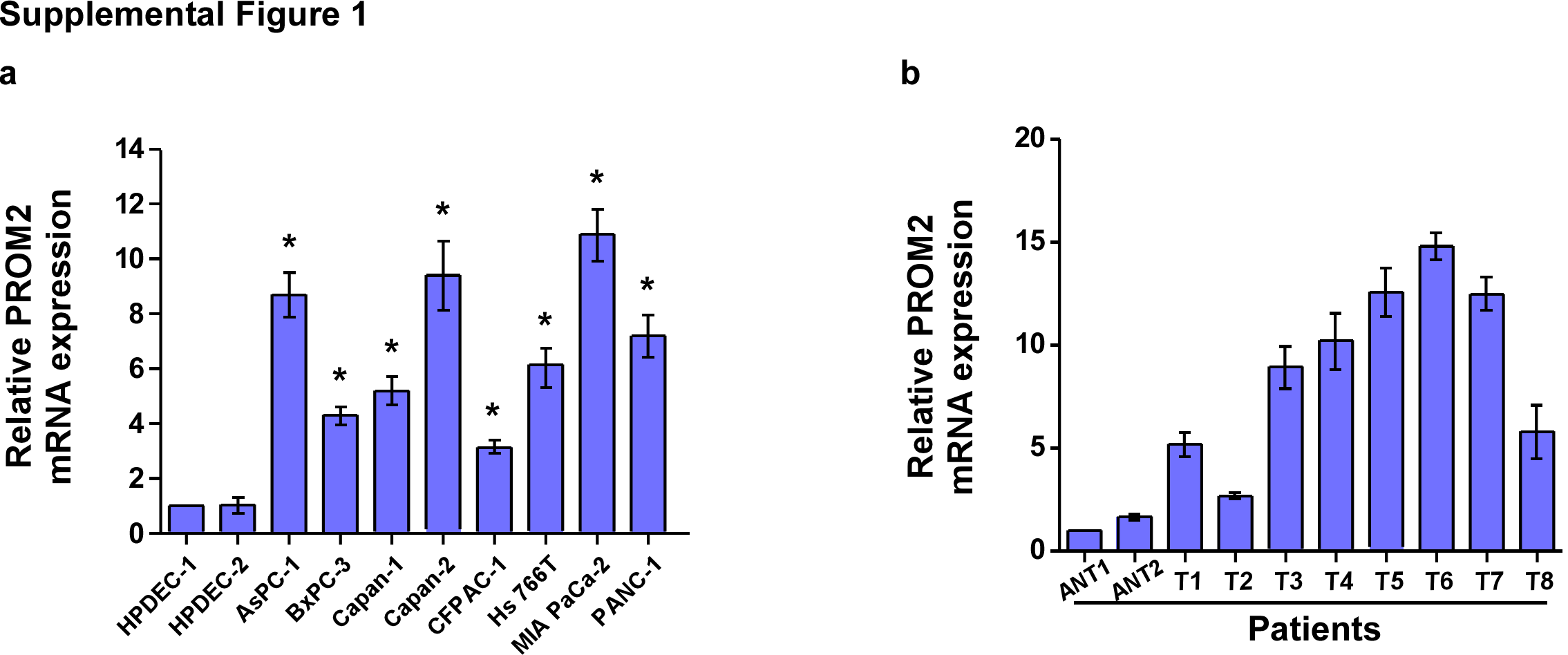


**Supplementary Fig. 1 PROM2 mRNA expression is upregualated in pancreatic cancer lines and tissues.** **a** PROM2 mRNA expression is upregulated in all 8 pancreatic cancer cell lines compared with immortal pancreatic ductal epithelial cell (HPDECs). **b** PROM2 protein is overexpressed in 8 pancreatic cancer tissues versus adjacent tissues of pancreatic cancer.


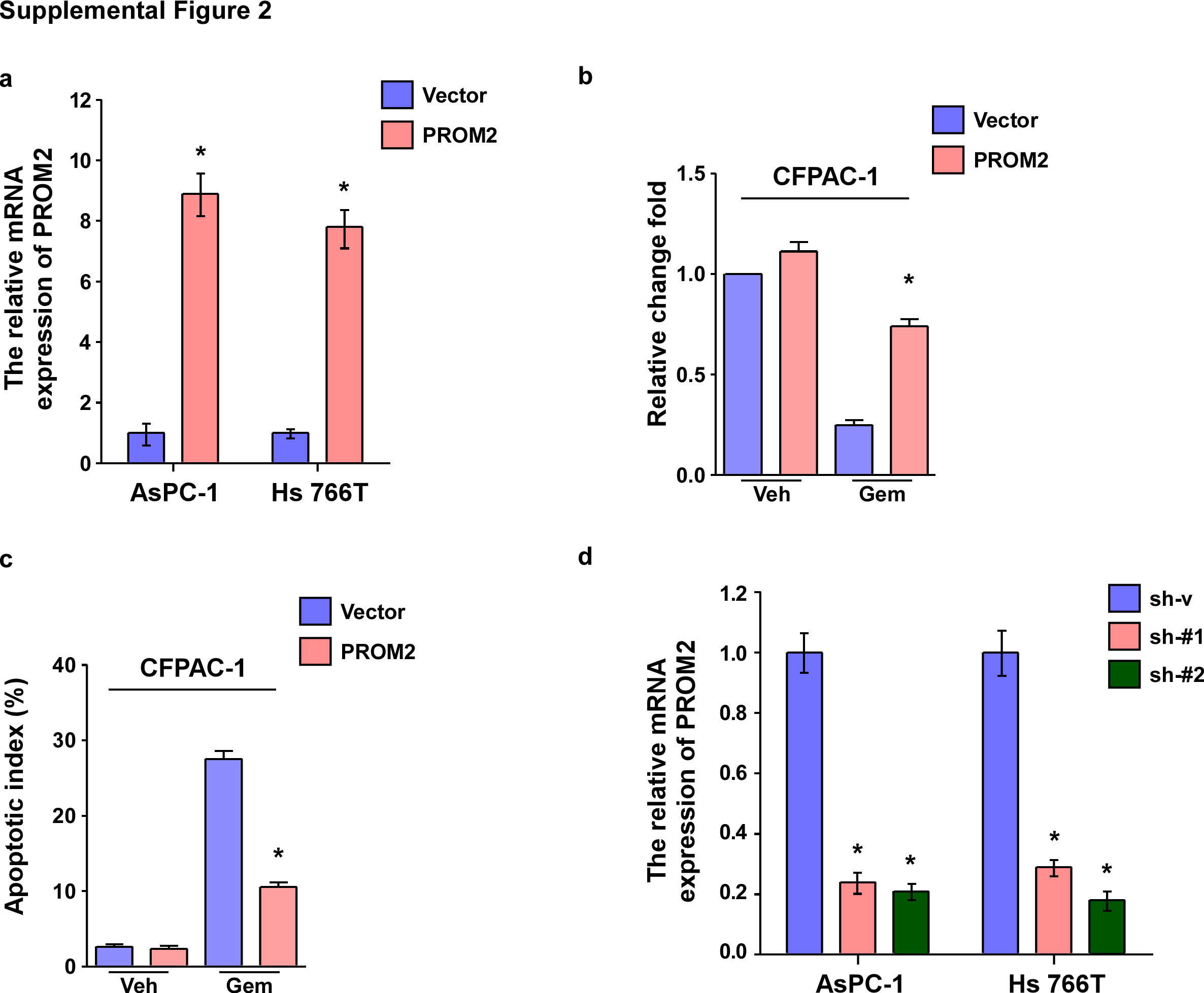


**Supplementary Fig. 2 PROM2 promotes gemcitabine resistance in pancreatic cancer cell lines.** **a** The relative mRNA expression of PROM2 is significantly increased in PROM2-overexprssed cells versus vector (* *P* < 0.05). The mRNA expression of vector group has been set as 1. **b** The relative mRNA expression of PROM2 is markedly reduced in PROM2-silenced cells versus scramble (* *P* < 0.05). The mRNA expression of scramble group has been set as 1. **c** The relative change fold of colony formation in indicated cells treated with vehicle or gemcitabine. **d** Apoptotic percentage in indicated cells treated with vehicle or gemcitabine.


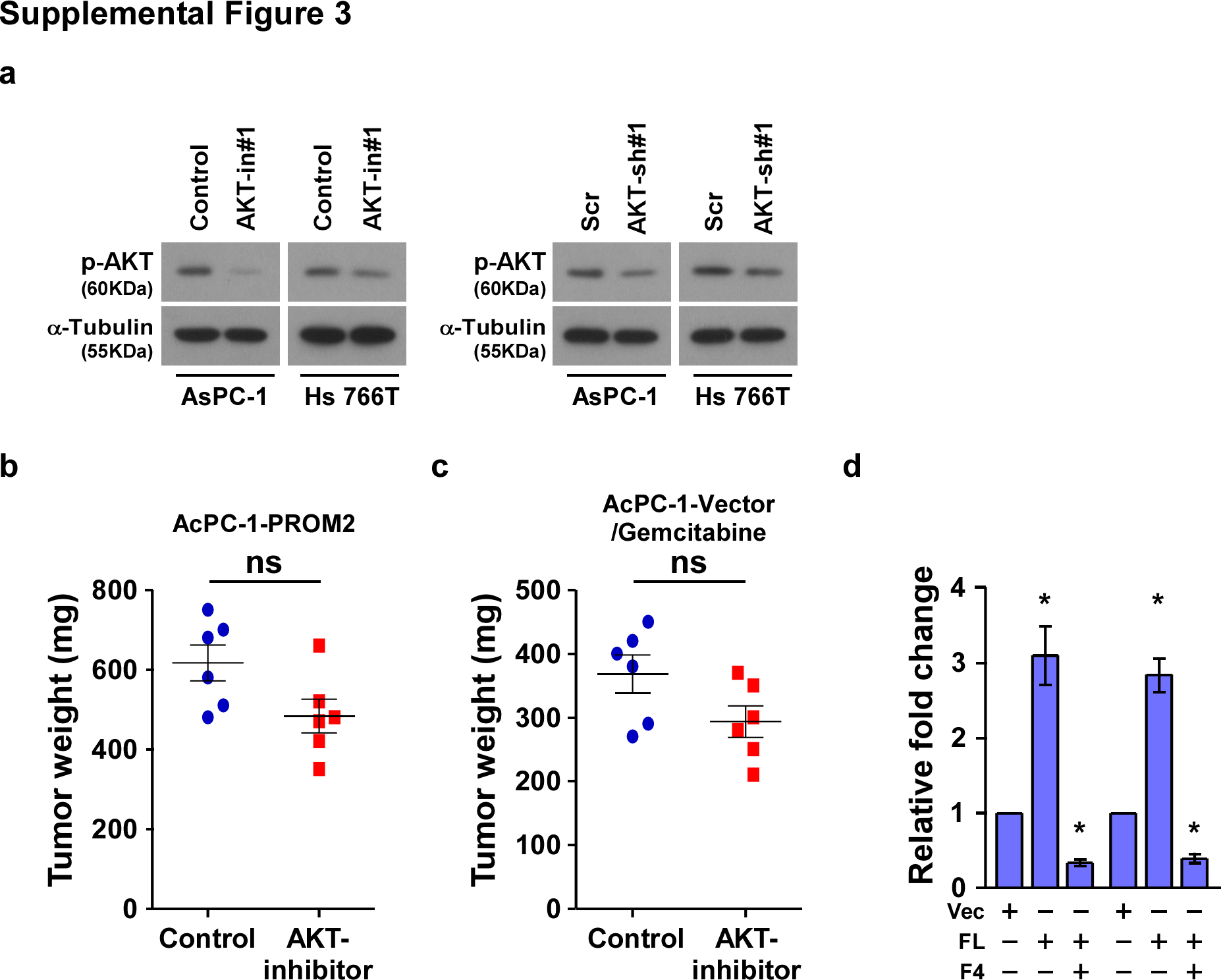


**Supplementary Fig. 3 Akt inhibitor restore gemcitabine sensitivity of pancreatic cancer cells. a** Immunoblotting assay illustrating the p-AKT levels of the indicated PROM2-overexpressing cells (Akt inhibitor MK2206, 5μM). **b** The tumor weight formed by AcPC-1/PROM2 cells in mice treated with control or Akt inhibitor MK2206. **c** The tumor weight formed by AcPC-1/Vector cells in mice treated with control or Akt inhibitor MK2206. **d** Quantification data (right) of colony numbers in the indicated cells treated with Gemcitabine (10μM) (mean ± SD, n = 3; * *P* < 0.05).
